# Determining the mechanical ventilation mode and pressure support combination that is best compatible with the rapid shallow breathing index calculated in spontaneous ventilation

**DOI:** 10.1186/cc13457

**Published:** 2014-03-17

**Authors:** S Demirtas Yilmaz, G Gürsel, M Aydogdu

**Affiliations:** 1 Gazi University Medical Faculty, Ankara, Turkey

## Introduction

Weaning from the mechanical ventilation (MV) composes about 40 to 50% of the total length of MV. Besides, there is no single reliable parameter indicating that patient will tolerate extubation safely. The rapid shallow breathing index (RSBI) is relatively the best predictive parameter for the initial assessment of readiness for discontinuation of MV support. But evaluation of the RSBI is valuable during T-tube ventilation; and in clinical practice it is not always possible to perform this assessment. In this study, we aimed to determine the best MV mode and pressure combinations that can predict successful RSBI closest to values calculated in spontaneous ventilation and estimate the patients' readiness for weaning.

## Methods

In this prospective cohort study, 25 mechanically ventilated patients were included. After 24 hours of MV, if the patients can successfully pass the daily screening test a spontaneous breathing trial (SBT) was initiated. RSBI and other weaning parameters were calculated in different combinations (PS:5 PEEP:5, PS:0 PEEP:5, PS:5 PEEP:0, PS:0 PEEP:0) before T-tube trial in all patients. Measurements in the spontaneous ventilation was performed with the COSMOPLUS Novometrix device that has both capnography and respiratory monitorisation function; other measurements were performed with ventilators.

## Results

The mean age of the study group was 73 ± 10 years; 11 of them were female and mean APACHE II score was 19 ± 6. RSBI did not differ significantly between spontaneous mode and other combinations, but the best correlation with spontaneous mode was found with PS:5 PEEP:0 (*P *= 0.0001, *r *= 0.719), and the worst with PS:0 PEEP:5 combination. RSBI calculated in each combination showed no predictive value for weaning success. Respiration rate (f) was higher in the SBT failure group than the SBT success group. When measured at PS:0 PEEP:5 and PS:5 PEEP:0 combinations, the threshold value of f was found to be 27/minute (*P *= 0.03).

## Conclusion

Although there was a correlation between RSBI measured in the T-tube and RSBI measured in different mode and pressure combinations, especially with the combination of PS:5 PEEP:0, a threshold value for RSBI cannot be detected during MV to predict SBT success.

